# Hepatic Enzyme Alterations in HIV Patients on Antiretroviral Therapy: A Case-Control Study in a Hospital Setting in Ghana

**DOI:** 10.1371/journal.pone.0134449

**Published:** 2015-08-06

**Authors:** Derick Nii Mensah Osakunor, Christian Obirikorang, Vincent Fianu, Isaac Asare, Mavis Dakorah

**Affiliations:** 1 Department of Molecular Medicine, School of Medical Sciences, College of Health Sciences, Kwame Nkrumah University of Science and Technology, Kumasi, Ghana; 2 Department of Medical Laboratory Technology, Faculty of Allied Health Sciences, Kwame Nkrumah University of Sciences and Technology, Kumasi, Ghana; 3 Medical Laboratory Department, St. Dominic Catholic Hospital, Akwatia, Ghana; University of Rome Tor Vergata, ITALY

## Abstract

**Background:**

Diagnosing hepatic injury in HIV infection can be a herculean task for clinicians as several factors may be involved. In this study, we sought to determine the effects of antiretroviral therapy (ART) and disease progression on hepatic enzymes in HIV patients.

**Methods:**

A case-control study conducted from January to May 2014 at the Akwatia Government Hospital, Eastern region, Ghana, The study included 209 HIV patients on ART (designated HIV-ART) and 132 ART-naive HIV patients (designated HIV-Controls). Data gathered included demography, clinical history and results of blood tests for hepatic enzymes. We employed the Fisher’s, Chi-square, unpaired t-test and Pearson’s correlation in analysis, using GraphPad Prism and SPSS. A *P* value < 0.05 was considered significant.

**Results:**

Median CD4 lymphocyte count of HIV-ART participants (604.00 *cells/mm^3^*) was higher than that of HIV-Controls (491.50 *cells/mm3; P* = 0.0005). Mean values of ALP, ALT, AST and GGT did not differ between the two groups compared (P > 0.05). There was a significant positive correlation between hepatic enzymes (ALP, ALT, AST and GGT) for both groups (p < 0.01 each). Duration of ART correlated positively with ALT (p < 0.05). The effect size of disease progression on hepatic enzymes for both groups was small.

**Conclusion:**

Antiretroviral therapy amongst this population has minimal effects on hepatic enzymes and does not suggest modifications in therapy. Hepatic injury may occur in HIV, even in the absence of ART and other traditional factors. Monitoring of hepatic enzymes is still important in HIV patients.

## Introduction

Antiretroviral Therapy (ART), a term that refers to the use of the combination of three or more antiretroviral agents, since its introduction, has dramatically altered the treatment and life expectancy of Human Immunodeficiency Virus (HIV) patients for the better [1, 2]. In spite of the benefits of ART, adverse effects [3], of which hepatotoxicity is a common finding can lead to discontinuation, switch and non-adherence to therapy [4].

Diagnosing hepatic injury in HIV infection can be a herculean task for clinicians, as several factors may be involved. That which includes HIV itself, hepatitis viruses (B and C), systemic opportunistic infections, malignancies and drug-induced hepatotoxicity [5].

Mechanisms of hepatotoxicity from ART may result from the interaction between anti-retroviral agents and other drugs metabolized in the liver. Dose-dependent toxicity, increased half lives (decreased clearance rate) of antiretroviral agents resulting from treatment with drugs like antibiotics and increase in the levels of anti-fungal agents to hepatotoxic levels by anti-retroviral agents themselves, are amongst other mechanisms [3].

Studies have shown that in patients commencing ART, 14–20% will experience elevations in hepatic enzymes [6]. Upon initiation of therapy however, most studies have found that mild hepatotoxicity exists amongst patients co-infected with hepatitis B or C virus [7–9] and after commencement of treatment for tuberculosis [10]. In Tanzania, efavirenz and rifampicin-based hepatotoxicity occurred in HIV patients with or without Tuberculosis (TB) co-infection but these were reported to be mild and did not require modifications in therapy [11].

Meanwhile, other researchers have reported an increased incidence of hepatic injury in ART-treated patients and have identified hepatotoxic events [4, 12, 13] including life-threatening episodes [14] in patients on ART. In Uganda, there is documented evidence of the absence of hepatotoxicity during ART [8].

These confirm that incidence rates of hepatotoxicity during ART may vary across different populations and with different drug combinations. Giving the different definitions used in different studies, the overall frequency of grade 3 or 4 liver toxicity induced by ART in HIV patients ranges from 1% to 18% [15, 16].

In Ghana, there was more than a 200-fold increase in the number of HIV patients receiving ART from 197 in 2003 to over 45,000 in 2010 [17]. With the increasing access to ART, there is the need to assess and monitor a plethora of co-morbidities that often present at the initiation or with the ageing of ART-treated patients.

To the best of our knowledge, this study is the first of its kind in Ghana. Thus, data on the extent of ART-related liver disease and the role of hepatic enzymes in monitoring this event, is limited.

In this study, we hypothesize that ART or HIV infection itself has hepatotoxic effects on HIV patients receiving treatment in Ghana. The purpose of this study was to determine the effect of ART and disease progression on hepatic enzymes, in a hospital setting in Ghana.

## Materials and Methods

### Study design/site

This case-control study was conducted at the Akwatia Government Hospital in the Eastern region of Ghana, from January to May 2014. The site is a district hospital that serves inhabitants of Akwatia and its surrounding areas.

### Study population

Three hundred and forty-one (341) participants were involved in the study, that which consisted of 209 patients on ART (designated HIV-ART) and 132 ART-naive patients (designated HIV-Controls).

Patients were included in the study if they met the following criteria; diagnosed and confirmed HIV-positive, age ≥ 18 years, followed up by the Akwatia Government Hospital with frequent visits, and on ART for at least three (3) months. Patients were excluded if they had active or chronic viral hepatitis, were pregnant, on concurrent hepatotoxic drugs or on treatment with liver supportive drugs.

### Ethical consent

The study was approved by the Committee on Human Research, Publication and Ethics (CHRPE) of the School of Medical Sciences (SMS), Kwame Nkrumah University of Science and Technology (KNUST) / the Komfo Anokye Teaching Hospital (KATH). Participation was voluntary and written informed consent was obtained from each participant.

### Data Collection and laboratory analysis

Demographic characteristics and clinical history were gathered using findings from a structured questionnaire and concurrent review of patient records.

About five (5) ml venous blood sample was taken from each participant, and dispensed into serum separator (SST) (3 ml) and ethylenediaminetetraacetic acid (EDTA) tubes (2ml). Serum biochemical assays [Alanine aminotransferase (ALT), Aspartate aminotransferase (AST), Alkaline Phosphatase (ALP) and Gamma-glutamyltransferase (GGT)] were performed immediately on the BT-3000 Plus Chemistry Analyser (Diamond Diagnostics, USA). CD4 lymphocyte count estimation was done on the BD FACS Count System (Becton Dickenson and Company, USA).

### Data management and analysis

Data was entered into a Microsoft Excel spread sheet and analysed using GraphPad Prism version 6.0 (GraphPad software, San Diego, California, USA) and Statistical Package for the Social Sciences (SPSS) version 20 (SPSS Inc. Chicago, USA).

Case definitions for the various hepatic enzymes were as follows; ALT > 40.0 U/L for male or > 31.0 U/L for female, AST > 37.0 U/L for male or > 31.0 U/L for female, GGT > 51.0 U/L for male or > 33.0 U/L for female and ALP > 117.0 U/L for adults [18]. Disease progression as indicated by CD4 lymphocyte count was; Stage1 (≥ 500 cells/mm^3^), Stage 2 (200–499 cells/mm^3^) and Stage 3 (< 200 cells/mm^3^) as per recommendations of the Centres for Disease Control and Prevention (CDC) [19–21]. Patients on ART were placed in three groups, based on duration of therapy; Group 1 (< 2 years), Group 2 (2–4 years) and Group 3 (> 4 years).

Categorical variables were expressed as frequencies and proportions and compared using the Fisher’s exact test or Chi-square test. Continuous variables were expressed as means ± SEM and compared using the unpaired t-test. To establish the relationship between variables, we performed the Pearson’s correlation co-efficient test. The Cohen's guidelines for eta squared was used in assessing the effect size of disease progression as follows; small = 0.02, medium = 0.06 and large = 0.13 [22]. A *P* value of < 0.05 was considered statistically significant.

## Results

Table 1 shows the demographic and clinical characteristics of the study population. Mean age was 40.85 ± 0.63 and majority were female (76.8%). Amongst the HIV-ART population, 45.5% had been on ART for less than 2 years. Median CD4 lymphocyte count of HIV-ART participants (604.00 *cells/mm* ^*3*^) was higher than that of HIV-Controls (491.50 *cells/mm3; P* = 0.0005). About half the population (50.1%) had CD4 counts ≥ 500 *cells/mm* ^*3*^ with a significant difference between proportions of the two groups (*P* < 0.0001).

**Table 1:** Demographic and clinical characteristics of study population.

Parameter	Total (n = 341)	HIV-ART (n = 209)	HIV-Controls (n = 132)	*P-value*
**Age (years)**				
Mean age	40.71 ± 0.63	41.05 ± 0.73	39.95 ± 1.27	0.4593
Young Adulthood (18–40 yrs)	184 (54.0)	119 (56.9)	65 (49.2)	0.3783
Middle Adulthood (41–65 yrs)	152 (44.6)	87 (41.6)	65 (49.2)	0.3783
Maturity (66 yrs—death)	5 (1.5)	3 (1.4)	2 (1.5)	0.3783
***Gender***				
Male	79 (23.2)	55 (26.3)	24 (18.2)	0.0882
Female	262 (76.8)	154 (73.7)	108 (81.8)	0.0882
***CD4 cell count (cells/mm*** ^***3***^ ***)***				
Median (IQR)	563.00 (362–928)	604.00 (384–4360)	491.50 (331–664)	**0.0005**
< 200	33 (9.7)	20 (9.6)	13 (9.8)	**< 0.0001**
200–499	137 (40.2)	113 (31.6)	24 (18.2)	**< 0.0001**
≥ 500	171 (50.1)	76 (58.9)	95 (72.0)	**< 0.0001**
**ART duration (years)**				
Median (IQR)	3.00 (1.7–4.0)	3.00 (1.7–4.0) -	-	-
Group 1 (< 2)	95 (27.9)	95 (45.5)	-	-
Group 2 (2–4)	74 (21.7)	74 (35.4)	-	
Group 3 **(>** 4)	40 (11.7)	40 (19.1)	-	
***ART regimen***				
AZT-3TC-EFV	48 (14.1)	48 (23.0)	-	-
AZT-3TC-LOP	7 (2.1)	7 (3.3)	-	
AZT-3TC-NVP	29 (8.5)	29 (13.9)	-	
AZT-EFV-NVP	9 (2.6)	9 (4.3)	-	
TDF-3TC-EFV	82 (23.3)	76 (36.4)	-	
TDF-3TC-NVP	27 (7.9)	27 (12.9)	-	
TDF-3TC-LOP	7 (2.1)	7 (3.3)	-	
TDF-3TC-EFV	6 (1.8)	6 (2.9)	-	

Data is presented as mean ± SEM or n (%), ART = Antiretroviral Therapy; TDF = Tenofovir Disoproxil Fumarate; FTC = Emtricitabine; LOP = Lopinavir/ritonavir, EFV = Efavirenz, AZT = Zidovudine; NVP = Nevirapine. P value < 0.05 was considered significant.

There was no significant difference in mean ALP (*P* = 0.8750), ALT (*P =* 0.6511), AST (*P =* 0.6128) and GGT (*P* = 0.0543) between the two groups (HIV-ART and HIV-Controls). [Table 2]

**Table 2:** Biochemical characteristics (hepatic enzymes) of study participants.

Parameters	HIV-ART (n = 209)	HIV- Controls (n = 132)	*P-value*
**ALP** (U/l)	125.4 ± 3.33	126.5 ± 6.11	0.8750
**ALT** (U/l)	24.85 ± 1.33	26.08 ± 2.36	0.6511
**AST** (U/l)	33.97 ± 1.64	35.82 ± 3.92	0.6128
**GGT** (U/l)	66.47 ± 4.47	89.51 ± 15.81	0.0543

Data is presented as mean ± SEM. ALP = Alkaline Phosphatase; ALT = Alanine aminotransferase; AST = Aspartate aminotransferase; GGT = Gamma-glutamyltransferase. P value < 0.05 was considered significant.

In Table 3, there was no significant difference between proportion of individuals with elevated hepatic enzymes, when compared between HIV-ART and HIV-Controls (P > 0.05).

**Table 3:** Participants with elevated hepatic enzymes.

Parameters	Total (n = 341)	HIV-ART (n = 209)	HIV-Controls (n = 132)	*P-value*
**ALP**	172 (50.4%)	106 (50.7%)	66 (50.0%)	0.9119
**ALT**	60 (17.6%)	34 (16.3%)	26 (19.7%)	0.4661
**AST**	113 (33.1%)	65 (31.1%)	48 (36.4%)	0.3454
**GGT**	226 (66.3%)	138 (66.0%)	88 (66.7%)	1.0000

Data is presented as n (%). ALP = Alkaline Phosphatase; ALT = Alanine aminotransferase; AST = Aspartate aminotransferase; GGT = Gamma-glutamyltransferase. P value < 0.05 was considered significant.

When this was compared within and across gender, all enzymes but the GGT, did not differ significantly between HIV-ART and HIV-Controls. Of both groups compared, there were significantly more males than females with elevated GGT (*P* < 0.0001 each). [Table 4]

**Table 4:** Comparison of hepatic enzyme levels between HIV-ART and HIV-Controls relative to gender.

Parameters	HIV-ART Male (n = 55)	HIV-Controls Male (n = 24)	*P value*	HIV-ART Female (n = 154)	HIV-Controls Female (n = 108)	*P-value*
***ALP***						
Normal	27 (49.0)	12 (50.0)	1.0000	76 (49.4)	54 (50.0)	1.0000
Elevated	28 (50.9)	12 (50.0)		78 (50.6)	54 (50.0)	
***ALT***						
Normal	49 (89.00	18 (75.0)	0.1700	126 (81.8)	88 (81.5)	1.0000
Elevated	6 (10.9)	6 (25.0)		28 (18.2)	20 (18.5)	
***AST***						
Normal	38 (69.1)	16 (66.7)	1.0000	106 (68.8)	68 (63.0)	0.3534
Elevated	17 (30.9)	8 (33.3)		48 (31.2)	40 (37.0)	
***GGT***						
Normal	32 (58.2) ***	16 (66.7)†††	0.6176	39 (25.3)	28 (25.9)	1.0000
Elevated	23 (41.8)	8 (33.3)		115 (74.7)	80 (74.1)	

Data is presented as n (%). ALP = Alkaline Phosphatase; ALT = Alanine aminotransferase; AST = Aspartate aminotransferase; GGT = Gamma-glutamyltransferase. P values indicate comparisons between HIV-ART males and HIV-Control males as well as between HIV-ART females and HIV-Control females. P value < 0.05 was considered significant.

* Indicate comparisons between males and females of HIV-ART).

^†^ Indicate comparisons between males and females of HIV-Controls).

***/††† is significant at the < 0.0001 level.

From Tables 5 and 6, there was a significant positive correlation between all hepatic enzymes (ALP, ALT, AST and GGT) for both the HIV-ART and HIV-Control groups (*P* < 0.01 each). Conversely, each of the enzymes is likely to rise concurrently with the rise of another. Duration of ART positively correlated with ALT (*P* < 0.05) but not with ALP, AST and GGT.

**Table 5:** Pearson’s correlation matrix showing the relationship between hepatic enzymes and duration of ART amongst HIV—ART participants.

Parameters	GGT	ALP	ALT	AST	ART duration
**GGT**		**0.46** **	**0.31** **	**0.42** **	**0.11**
**ALP**			**0.44** **	**0.48** **	**0.01**
**ALT**				**0.76** **	**0.15** *
**AST**					**0.13**

Correlation is significant at

* P < 0.05 and

** P < 0.01 (2-tailed).

ART = Antiretroviral Therapy; ALP = Alkaline Phosphatase; ALT = Alanine Transaminase; AST = Aspartate Transaminase; GGT = Gamma-glutamyltransferase.

**Table 6:** Pearson’s correlation matrix of hepatic enzymes amongst the HIV—Control group.

Parameters	GGT	ALP	ALT	AST
**GGT**		**0.41** **	**0.40** **	**0.86** **
**ALP**			**0.45** **	**0.51** **
**ALT**				**0.53** **
**AST**				

Correlation is significant at

* P < 0.05 and

** P < 0.01 (2-tailed).

ART = Antiretroviral Therapy; ALP = Alkaline Phosphatase; ALT = Alanine Transaminase; AST = Aspartate Transaminase; GGT = Gamma-glutamyltransferase.

Table 7 presents the relationship between disease progression, as indicated by the CD4 lymphocyte counts and ART-related hepatic injury. The effect size of disease progression on hepatic enzymes for the HIV-ART group was small, as indicated by eta squared for the hepatic enzymes. This was similar for the HIV-Control group.

**Table 7:** Relationship between disease progression and ART-related hepatic injury.

	Parameters	Stage 1	Stage 2	Stage 3	Eta Squared	*P* for Trend
**HIV-Controls**	**ALP** (U/l)	126.84 ± 7.43	138.05 ± 14.07	107.67 ± 8.12	0.045	0.2380
	**ALT** (U/l)	24.19 ± 1.78	27.64 ± 5.67	29.27 ± 6.54	0.012	0.6970
	**AST** (U/l)	38.97 ± 6.84	33.86 ± 6.17	33.08 ± 5.41	0.007	0.7980
	**GGT** (U/l)	101.33 ± 29.35	76.96 ± 20.93	80.00 ± 21.22	0.008	0.7730
**HIV-ART**	**ALP** (U/l)	123.36 ± 4.58	131.84 ± 6.42	131.54± 13.98	0.008	0.5310
	**ALT** (U/l)	23.18 ± 1.59	25.66 ± 2.34	24.69 ± 4.36	0.005	0.6610
	**AST** (U/l)	32.13 ± 1.99	33.63 ± 2.64	33.54 ± 5.94	0.001	0.8930
	**GGT** (U/l)	60.63 ± 5.09	78.64 ± 11.44	61.31 ± 12.70	0.016	0.2700

Data presented as mean ± SEM. ART = Antiretroviral Therapy; ALP = Alkaline Phosphatase; ALT = Alanine Transaminase; AST = Aspartate Transaminase; GGT = Gamma-glutamyltransferase; Disease progression through categorization of CD4 count: Stage 1 represents ≥ 500/mm^3^, Stage 2 represents 200–499/mm^3^ and Stage 3 represents < 200/mm^3^.

## Discussion

In spite of the benefits of ART, it may come with adverse effects [3], hence the question of discontinuation or alteration of treatment may come to play [4]. In this study, we sought to determine the effect of ART and disease progression on hepatic enzymes, in a hospital setting in Ghana.

The results of the present study suggest that in this population, ART has minimal effects on hepatotoxicity. This may be due to the absence of other traditional risk factors such as hepatitis C virus and / or hepatitis B virus (HBV / HCV) co-infection, older age, high alcohol intake and use of illicit drugs that may contribute to the development of hepatic injury in adults [23, 24].

Upon HIV infection, it is expected that CD4 lymphocyte counts will drop with ageing of HIV infection, and ART is expected to help increase these numbers. We observed that the median CD4 count of HIV-ART participants was higher than that of HIV-Controls. Findings from other researchers have shown that, after ART, the median CD4 cell counts increase with time [25]. Adherence to therapy may also be a contributing factor to achieving the purpose of therapy in HIV patients [26], thus the finding in the present study. However, since we were unable to assess adherence to therapy, we are unable to ascertain this fact. Recently, Obirikorang *et al*., [27] found a lower CD4 lymphocyte count amongst HIV patients on ART than in those patients who were not. They attributed this to the fact patients who were not on ART had values not so low as to initiate ART, hence causing a possible masked increase in CD4 lymphocyte count amongst patients on ART when compared to ART-naive individuals [27]. Hence, the suggestion of an increase in CD4 lymphocyte count upon ART initiation is still valid.

Hepatic enzyme elevations are common in HIV-infected patients, especially those treated with ART. Despite such reports, HIV-infected patients may present several risk factors for biochemical abnormalities, and a precise aetiology is rarely defined clearly [28]. Most studies conducted on hepatotoxicity have shown the existence of hepatic injury associated with ART, but in the presence of other co-infections such as HCV [7] and even tuberculosis [11]. In a retrospective study assessing severe hepatic injury during ART, co-infected patients had a 3.99 greater chance of developing severely elevated transaminase levels compared with patients not co-infected with HCV. This risk increased considerably in co-infected patients with identified alcohol abuse [23]. We observed that of all the hepatic enzymes measured (ALT, AST, ALP and GGT), there was no significant difference in mean values between the two groups compared (HIV-ART and HIV-Controls). Thus in the absence of other traditional risk factors that increase the chances or cause hepatotoxicity, injury by ART may be minimal. On the contrary, a study conducted in Cameroon found elevations in both serum AST and ALT in patients on ART [13].

Reports have shown that slight increases in GGT may be due to contribution from other sources of GGT such as the prostate, pancreas and kidneys [29, 30]. In the case of the current study, higher proportion of males with elevated levels of GGT observed could be due in part, to contributions from extra-hepatic sources such as the prostate.

The observed small effect size of disease progression on hepatic enzymes could indicate that with careful studies, there is indeed an effect of disease progression on hepatic enzymes in HIV but this may not be clinically obvious. Mokondjimobe *et al*., [31] reported that isolated naive HIV infection was associated with higher levels of ALT and GGT before and after adjusting for age and sex. Other researchers have also suggested that in the absence of ART, mild hepatotoxicity is seen in HIV patients [32]. This has been attributed to the fact that HIV infection results from chronic immune system activation and inflammatory cytokine release and studies have shown that ALT and GGT are now considered markers of inflammation with immune dysfunction in HIV [33] and oxidative stress [34]. As disease progresses and chronic inflammation increases, there is a high likelihood of increases in these hepatic enzymes.

The present study is limited by a number of factors. Our inability to determine the HIV viral RNA in study participants could not enable the determination of the relationship between viral load, ART regimen and liver enzymes, and thus the role of HIV itself on changes in liver enzymes. Furthermore, information on the number of ART regimens used by participants was not available, hence the effects of the number of regimens on liver enzymes was not determined. As this is a case control study, we were unable to determine pre-treatment liver enzyme levels and subsequently follow up with post-treatment levels. The inclusion of higher number of participants, information on antiretroviral exposure, increased observational time, and the use of a more restrictive criterion with regards to antiretroviral exposure is warranted.

## Conclusion

Antiretroviral therapy amongst this population has minimal effects on hepatic enzymes and does not suggest modifications in therapy. In the absence of traditional risk factors for hepatotoxicity like alcohol use, viral hepatitis and illicit drug use, hepatic injury may be caused by HIV infection itself. This may however be seen only upon careful studies. Frequent measurement of hepatic enzymes is still important for HIV patients on and off ART, with and without other traditional risk factors for hepatotoxicity.
